# A short-term, hydroponic-culture of ginseng results in a significant increase in the anti-oxidative activity and bioactive components

**DOI:** 10.1007/s10068-020-00735-5

**Published:** 2020-03-07

**Authors:** Ji Yun Lee, Hee Yang, Tae Kyung Lee, Chang Hyung Lee, Ji Won Seo, Jong-Eun Kim, Seo Yeong Kim, Jung Han Yoon Park, Ki Won Lee

**Affiliations:** 1 Department of Agricultural Biotechnology and Research Institute of Agriculture and Life Sciences, Seoul National University, Seoul, 08826 Republic of Korea; 2 Advanced Institutes of Convergence Technology, Seoul National University, Suwon, 16229 Republic of Korea; 3 Department of Food Science and Technology, Korea National University of Transportation, Jeungpyeong, 27909 Republic of Korea

**Keywords:** *Panax ginseng* CA Meyer, Hydroponic cultivation, Ginseng, Ginsenosides, Phenolic compounds

## Abstract

*Panax ginseng* CA Meyer has a variety of biological effects, including antioxidant and antidiabetic activities. Ginseng requires long-term cultivation, but this can be shortened using hydroponic systems to facilitate the commercial development of ginseng as a functional food. However, the characteristics of short-term-cultured (< 30 days) hydroponic ginseng (sHCG) are unclear. We investigated the characteristics of 21-day-cultured sHCG compared 5-year-old normally cultured ginseng. The free radical-scavenging activity and total ginsenoside and phenolic contents were significantly higher in sHCG than in normally cultured ginseng. Fifteen ginsenosides were detected in sHCG, and the concentrations of most were higher in shoots than roots. These findings suggest that 21-day-cultured sHCG, due to its enhanced antioxidant activity and higher concentrations of total phenolics and ginsenosides (including Rd and Re), has potential as a functional food.

**Electronic supplementary material:**

The online version of this article (10.1007/s10068-020-00735-5) contains supplementary material, which is available to authorized users.

## Introduction

*Panax ginseng* CA Meyer (Korean ginseng) is a perennial plant of the family *Araliaceae* and has been used as an ingredient in traditional herbal medicines for over 2,000 years, particularly in Asia. Korean ginseng is currently distributed to 35 countries worldwide (Jung et al., 2002).

Ginseng exerts various bioactive effects, including antioxidant and antitumor activities. Prior studies have focused on saponins (ginsenosides), as the primary active ingredients in ginseng, because saponins exert several beneficial effects (Christensen, 2009). The total ginsenoside content is an important determinant of the potency of ginseng. To date, more than 100 ginsenosides have been identified in *P. ginseng* (Shin et al., 2015). most of which belong to one of two major functional types: protopanaxadiol (PPD) and protopanaxatriol (PPT) types. As these two types of ginsenoside exert different physiological effects (Chen et al., 2016), the PPD/PPT ratio is calculated to determine the bioactivity of ginseng (Shan et al., 2014).

The commercial application of ginseng root has been a focus of research. However, other parts of the *P. ginseng*, such as the shoot, which includes the leaf and stem, also contain a variety of ginsenosides (Li et al., 1996). Leaves have a higher ginsenoside content than that of primary roots cultivated for the same duration (Kang and Kim, 2016). Most ginsenosides accumulate in leaves during the early growth stages, i.e., the first and second years (Shi et al., 2007).

Ginseng used for commercial purposes is harvested after cultivation for at least 5–6 years (Soldati and Tanaka, 1984). Recently, a method of short-term cultivation (~ 120 days) of ginseng seedlings in hydroponic systems has been developed to facilitate the commercialization of *P. ginseng* as a functional food (Kim et al., 2012). In hydroponic systems, crops are cultivated in nutrient solution instead of soil and in the absence of crop-protective agents such as pesticides. In such systems, it is easier to control key factors in the growth environment including temperature, light intensity, and moisture. Hydroponic cultivation produces higher concentrations of ginsenosides in leaves and roots over a shorter period (Choi et al., 2012). However, although several studies have focused on hydroponic ginseng cultured for 90–120 days, few have evaluated culture durations of less than 30 days. After transplantation of 2-year-old ginseng into a hydroponic system, the ginsenoside content of leaves steadily increased from 7 to 56 days, whereas that in roots increased until 21 days and steeply declined thereafter. Therefore, the optimal cultivation duration for 2-year-old ginseng seedlings after transplantation in hydroponic systems may be less than 30 days (Jang et al., 2018).

We evaluated the antioxidant activity, total ginsenoside content, phenolic content and profiles of 22 ginsenosides in 21-day hydroponically cultured ginseng (sHCG) compared with 5-year-old normally cultured ginseng.

## Materials and methods

### Sample preparation

One-year-old ginseng seedling roots (*Panax ginseng* CA Meyer) were purchased from Geumsan Ginseng National Agricultural Cooperative Federation (Geumsan, Republic of Korea [ROK]) after cultivation in a greenhouse. To obtain sHCG, the seedling roots were transplanted into nutrient baths and cultured in a hydroponic system at 22 °C under mixed red and blue LED light. A mineral nutrient solution (Farmcraft, Gimpo, ROK) was sprayed onto the roots twice daily. After culturing for 21 days, the sHCG was harvested and used whole or separated into shoots (sHCG-S) and roots (sHCG-R). Normally cultured (5-year-old) ginseng roots were obtained from the Anseong Ginseng National Agricultural Cooperative Federation (Anseong, ROK). The ginseng samples were washed with distilled water and stored at − 70 °C. The samples were lyophilized and ground into powder using a blender (Shinil, Cheonan, ROK). The lyophilized powder was extracted as described previously (Chung et al., 2016a). Briefly, the powder (0.5 g) was extracted with 50 mL 80% methanol by mixing at 220 rpm at room temperature overnight. The extract was centrifuged at 10,000×*g* for 20 min, and the supernatant was filtered through a 0.2 μm syringe filter and evaporated. The residue was lyophilized and stored at − 70 °C until use.

### Total antioxidant activity assay

The total antioxidant activity was evaluated by 1,1-diphenyl-2-picrylhydrazyl (DPPH) radical scavenging assay (Lee et al., 2017). Antioxidant activity is expressed as milligrams of vitamin C equivalent antioxidant capacity (VCEAC) per 100 g dry weight.

### DCF-DA assay

Antioxidant activity against hydrogen peroxide (H_2_O_2_)-induced intercellular ROS was evaluated by DCF-DA assay using 1 mM H_2_O_2_ and HaCaT cells, as described by Cho et al. (2002) and Yoon et al. (2017) with modifications. ROS accumulation was measured at an emission wavelength of 530 nm and an excitation wavelength of 485 nm after incubation for 30 min in DCF-DA at 25 °C. The fluorescence signal was measured using a microplate reader (Tecan M200 Pro, Männedorf).

### Analysis of ginsenosides

Chromatographic separation was achieved using the LaChromUltra L-2000 U-Series ultra-high-performance liquid chromatograph (ultra-HPLC; Hitachi-High Technologies, Ibaraki-ken, Japan). The HPLC conditions, including the linear mobile phase gradient, column, and detection wavelength, were as described previously (Ha et al., 2013). A total of 22 ginsenosides were analyzed by ultra-HPLC, including 12 PPD types [Rd, Rb2, Rc, Rb1, Rb3, F2, Rg3(S), Rg3(R), K, Rh2(S), Rh2(R), and PPD] and 10 PPT types [Re, Rg1, F1, Rh1(S), Rh1(R), Rg2(S), Rg2(R), Rf, PPT(S), and PPT(R)]. The PPD/PPT ratio was calculated as the total PPD content divided by the total PPT content.

### Total phenolic content assay

The total phenolic content was determined by Folin–Ciocalteu assay with modifications (Lee et al., 2017). Activity was expressed as milligrams of catechin equivalents (CE) per 100 g dry weight.

### Analysis of ginsenosides Rd, Rb1, Re and Rg1

The four major ginsenosides (Rd, Rb1, Re, and Rg1) were analyzed by high performance liquide chromatography with diode array detector (HPLC–DAD) as described previously (Lau et al., 2003). Briefly, 1 g sample was extracted with 10 mL 70% (v/v) aqueous methanol for 2 h. The extracted solution was centrifuged (850 × g, 10 min, 4 °C), filtered through a 0.22 μm nylon membrane, and analyzed on the Ultimate 3000 HPLC system (Thermo Dionex) using a 4.6 × 250 mm, 5 μm Inno C-18 column (Youngjin Biochrom, ROK). The column temperature was maintained at 30 °C. Solvent A was 0.3% TFA, and solvent B was acetonitrile. The flow rate was 1 mL/min, and the following elution gradient was applied: 0–2 min, 75% A; 2–15 min, 75–60% A; 15–16 min, 60–0% A; 16–20 min, 0% A; 20–21 min, 0-75% A; 21–28 min, 75% A. Ginsenosides were detected at 215 nm. The levels of the four ginsenosides in ginseng were quantified using an external standard curve (R^2^ > 0.99).

### Statistical analysis

Data are expressed as mean ± standard deviation. The significance of the differences between mean values was determined by Student’s *t* test. A value of *p* < 0.05 or < 0.01 was used as the criterion for statistical significance.

## Results and discussion

### Bioactive component contents

**Fig. 1:**
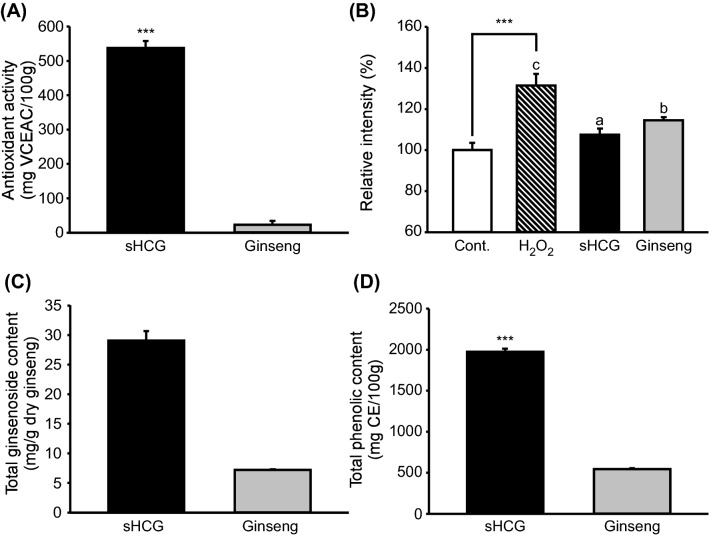
Characteristics of sHCG and ginseng. 2,2-Diphenyl-1-picrylhydrazyl (DPPH) [if you define one abbreviation then define them all, as done here] antioxidant activity (**A**), effects of sHCG and ginseng on the intracellular ROS levels of H_2_O_2_-treated HaCaT cells (**B**), and the total ginsenoside (**C**) and total phenolic (**D**) contents of sHCG and ginseng. Data are means ± SD (n = 2–4). sHCG, short-term hydroponically cultured ginseng

The medicinal effects of ginseng are linked to its antioxidant activity (Fu and Ji, 2003). The free radical-scavenging activity, as determined by DPPH assay, was approximately 23-fold higher for sHCG (537.3 ± 21.1 mg VCEAC/100 g) than normally cultured ginseng (23.2 ± 11.1 mg VCEAC/100 g) (Fig. 1A). The intracellular antioxidant activities of sHCG and ginseng were evaluated by DCF-DA. To induce ROS production, HaCaT cells were treated with H_2_O_2_ (1 mM), a free radical (Wu et al., 1996). The ROS level in H_2_O_2_-treated cells was significantly increased, and that of sHCG-treated cells was significantly decreased, compared with ginseng-treated cells (Fig. 1B). These results were similar to those obtained by DPPH assay. Various ginsenosides have antioxidant activity (Sodrul et al., 2018), and thus we examined the total ginsenoside content of sHCG and ginseng. The total ginsenoside content of sHCG (29.0 mg/g dry ginseng) was more than fourfold that of ginseng (7.2 mg/g dry ginseng) (Fig. 1C). In addition to saponins, non-saponin compounds including phenolic acids contribute to the antioxidant activity of ginseng (Chung et al., 2016b). Folin–Ciocalteu assays revealed a significantly higher total phenolic content in sHCG (1975.9 ± 37.5 mg CE/100 g) than normally cultured ginseng (543.6 ± 11.2 mg CE/100 g) (Fig. 1D). These findings suggest that sHCG has stronger antioxidant activity than that of ginseng, which may be attributed to its higher contents of ginsenosides and phenolics.

### Bioactive components in sHCG shoots and roots

**Fig. 2:**
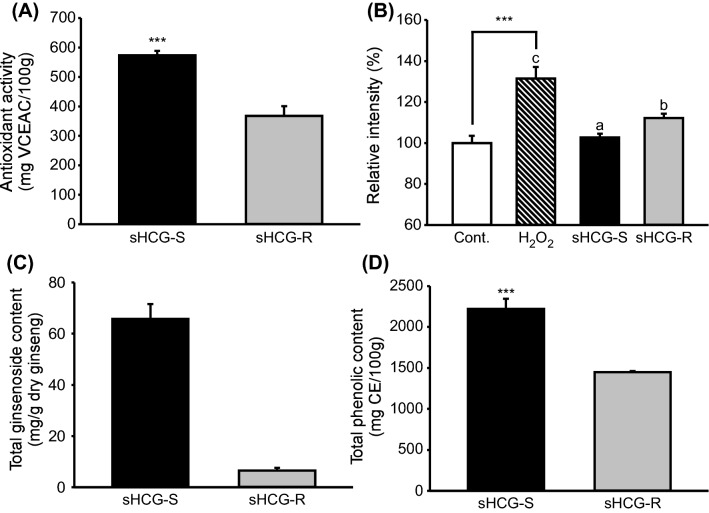
Characteristics of sHCG-S and sHCG-R. DPPH antioxidant activity (**A**), effects of sHCG-S and sHCG-R on the intracellular ROS levels of H_2_O_2_-treated HaCaT cells (**B**), and the total ginsenoside (**C**) and total phenolic (**D**) contents of sHCG-S and sHCG-R. Data are means ± SD (n = 2–3). sHCG-S, short term hydroponically cultured ginseng shoot; sHCG-R, short-term hydroponically cultured ginseng root

After hydroponic cultivation of 1-year-old ginseng seedlings for 21 days, sHCG was divided into sHCG-S and sHCG-R. The dry weight ratio of the two parts was 0.8:1 (data not shown). When sHCG-S and sHCG-R were calculated based on this ratio, the result was 29.55 mg/g dry ginseng. The total amount of sHCG was 29.03 mg/g dry weight. Therefore, the total amount of sHCG was similar to calculated sHCG-S and sHCG-R. Shoots and roots have different compositions, including ginsenosides and phenolic compounds (Chung et al., 2016b). The antioxidant activity was significantly higher in sHCG-S (573.1 ± 15.2 mg VCEAC/100 g) than in sHCG-R (367.5 ± 32.8 mg VCEAC/100 g) (Fig. 2A). The antioxidant activities of sHCG-S and sHCG-R were evaluated by DCF-DA. To induce ROS production, HaCaT cells were treated with H_2_O_2_ (1 mM). The ROS level was significantly lower in HaCaT cells treated with sHCG-S than in those treated with sHCG-R (Fig. 2B), similar to the results of the DPPH assays. The total ginsenoside content was approximately 10-fold higher in sHCG-S (65.7 mg/g dry ginseng) than in sHCG-R (6.5 mg/g dry ginseng) (Fig. 2C). The total phenolic content was also significantly higher in sHCG-S (2223.4 ± 120.1 mg CE/100 g) than in sHCG-R (1447.4 ± 13.9 mg CE/100 g) (Fig. 2D). These results indicate that sHCG-S contains more ginsenosides and phenolic compounds than does sHCG-R, leading to higher antioxidant activity. These findings are consistent with previous studies of aged ginseng specimens (Zhang et al., 2014).

### Profiles of ginsenosides in sHCG, sHCG-S, and sHCG-R

**Fig. 3:**
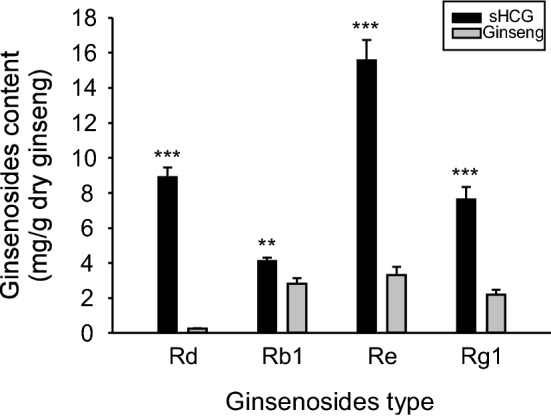
Contents of four ginsenosides in sHCG and ginseng. Rd, Rb1, Re, and Rg1 contents of sHCG and ginseng (mg/g dry ginseng). Data are means ± SD (n = 3). sHCG, short term hydroponically cultured ginseng

Of the 22 ginsenosides analyzed, 15 were detected in the original profiling. The Rd content of PPD type and the Re content of PPT type ginsenosides were highest in the sHCG profile (Supplementary data 1). Therefore, we analyzed Rd and Re together with Rb1 and Rg1, marker compounds of ginseng. The levels of these four ginsenosides were significantly higher in sHCG than in ginseng (Fig. 3). The Rd content was 36.4-fold higher in sHCG (8.89 mg/g dry ginseng) than ginseng (0.24 mg/g dry ginseng) and the Re content 4.7-fold higher in sHCG (15.55 mg/g dry ginseng) than in ginseng (3.30 mg/g dry ginseng). The Rb1 and Rg1 contents were 1.5- and 3.5-fold higher in sHCG (4.10 and 7.61 mg/g dry ginseng) than in ginseng (2.80 and 2.18 mg/g dry ginseng), respectively. It is speculated that the ginsenoside components present in sHCG at high levels are determinants of its antioxidant activity; therefore, further studies are warranted.

Here, we report for the first time higher antioxidant capacity and total phenolic and ginsenoside contents in that 21-day cultured sHCG compared with 5-year-old normally cultured ginseng. These findings indicate that shortening the cultivation period can increase the contents of functional compounds, which may be aided by LED light stimulation. Moreover, smart-farming systems have emerged to replace plant factories, permitting automation and optimization of plant growth. Further studies are needed to determine the components responsible for the biological effects of ginseng—both ginsenosides and other compounds, such as phenolics and polysaccharides—and the environmental factors that can be modulated to further increase the contents of these components of interest.

## Electronic supplementary material

Below is the link to the electronic supplementary material.

Supplementary material 1 (DOCX 81 kb)
