# Supplementary material for: A short-term, hydroponic-culture of ginseng results in a significant increase in the anti-oxidative activity and bioactive components
Source: Food Sci Biotechnol. 2020 Mar 7;29(7):1007–12. doi: 10.1007/s10068-020-00735-5 (PMC7297876; doi:10.1007/s10068-020-00735-5)
Supplement: Supplementary file 1 — Supplementary material 1 (DOCX 81 kb) [file 10068_2020_735_MOESM1_ESM.docx]

**Materials and Methods**

**Analysis of ginsenosides** Chromatographic separation was achieved by using a LaChromUltra L-2000 U-Series apparatus (Hitachi-High Technologies, Japan). HPLC conditions including linear solvent gradient of mobile phases, column, and wavelength of detector were applied according to a previous study ([Ha, Shim et al. 2013](#_ENREF_1)). A total of 22 ginsenosides were analyzed in an ultra-HPLC system, which included 12 PPD types [Rd, Rb2, Rc, Rb1, Rb3, F2, Rg3(S), Rg3(R), K, Rh2(S), Rh2(R), PPD] and 10 PPT types [Re, Rg1, F1, Rh1(S), Rh1(R), Rg2(S), Rg2(R), Rf, PPT(S), PPT(R)]. The PPD/PPT ratio was calculated as total PPD divided by total PPT types.

**References**

1. Ha, J., Y. S. Shim, D. Seo, K. Kim, M. Ito and H. Nakagawa. Determination of 22 ginsenosides in ginseng products using ultra-high-performance liquid chromatography. *J Chromatogr Sci.* 51(4): 355-360 (2013)

**Supple T1.** **Profiling of ginsenosides in sHCG, sHCGS, sHCGR and ginseng.** sHCG, short term hydroponic-cultured ginseng; short term

hydroponic-cultured ginseng shoot; sHCG-R, short term hydroponic-cultured ginseng root. Data are expressed as a mean $\pm$ SD (n=2). ND,

not detectable.
